# A short-interval longitudinal study of associations between psychological distress and hippocampal grey matter in early adolescence

**DOI:** 10.1007/s11682-023-00847-6

**Published:** 2024-01-13

**Authors:** Amanda Boyes, Jacob M. Levenstein, Larisa T. McLoughlin, Christina Driver, Lia Mills, Jim Lagopoulos, Daniel F. Hermens

**Affiliations:** Thompson Institute, UniSC, 12 Innovation Parkway, Birtinya, QLD 4575 Australia

**Keywords:** Mental health, Adolescence, Psychological distress, Hippocampus, Grey matter volume

## Abstract

This study of Australian adolescents (*N* = 88, 12-13-years-old) investigated the relationship between hippocampal grey matter volume (GMV) and self-reported psychological distress (K10) at four timepoints, across 12 months. Participants were divided into two groups; those who had K10 scores between 10 and 15 for all four timepoints were categorised as “low distress” (i.e., control group; *n* = 38), while participants who had K10 scores of 16 or higher at least once over the year were categorised as “moderate-high distress” (*n* = 50). Associations were tested by GEE fitting of GMV and K10 measures at the same time point, and in the preceding and subsequent timepoints. Analyses revealed smaller preceding left GMV and larger preceding right GMV were associated with higher subsequent K10 scores in the “moderate-high distress” group. This was not observed in the control group. In contrast, the control group showed significant co-occurring associations (i.e., at the same TP) between GMV and K10 scores. The “moderate-high distress” group experienced greater variability in distress. These results suggest that GMV development in early adolescence is differently associated with psychological distress for those who experience “moderate-high distress” at some point over the year, compared to controls. These findings offer a novel way to utilise short-interval, multiple time-point longitudinal data to explore changes in volume and experience of psychological distress in early adolescents. The results suggest hippocampal volume in early adolescence may be linked to fluctuations in psychological distress.

**Supplementary Information:**

The online version contains supplementary material available at 10.1007/s11682-023-00847-6.

## Introduction

Adolescence involves rapid biological, social, emotional, and cognitive changes, and unique health and developmental needs (Blakemore, 2012; Tetzner et al., 2017; World Health Organisation, 2014). Moreover, altered brain development trajectories have been linked to the onset and development of psychiatric symptoms in adolescents (Bick & Nelson, 2016; Whittle et al., 2013). A survey of mental health and wellbeing in Australia found that 39.6% of people aged 16–24 reported experiencing symptoms of a diagnosed disorder in the last 12 months, the highest rate of any decade of life (Australian Bureau of Statistics, 2022). Further, people aged 16–34 were most likely to experience high or very high levels of psychological distress (PD; rate 20.0%) (Australian Bureau of Statistics, 2022). PD is an indication of current emotional experience, typically relating to anxious and depressive symptoms, with measures indicating likelihood of a current or future mental illness (Welsh et al., 2020). Thus, it is important to understand how biological and psychological factors interact in adolescence, to create effective early interventions and improve young people’s mental health outcomes.

PD measures may identify opportunities to develop resilience to mental health disorders prior to onset, and there is overlap of emerging symptoms and risk factors across clinical phenotypes (Mennigen & Bearden, 2020). Anxiety and depression emerge during adolescence, which is also a key developmental period for grey matter (Bethlehem et al., 2022). Thus, identifying potential changes in neurobiology, and/or how these changes are associated with mental health symptoms prior to the onset of a disorder, in real-time, may provide insights for interventions. The Kessler-10 (K10) is useful in screening for psychological disorders including depression and anxiety, and scores are significantly related to the presence of serious mental illness (Kessler et al., 2003; Lawrence et al., 2015; Sunderland et al., 2011). Genetics and environmental factors influence brain structure over our lifespan, and life experiences, including stress and childhood/adolescent onset depression, can be linked to both adolescent neurobiology and mental health (Bethlehem et al., 2022; Luby et al., 2016; Miguel et al., 2019; Tooley et al., 2021; Whittle et al., 2014). The K10 captures current experience of PD, and it one of the most widely-used and well-validated screening tools for psychological symptoms and distress (Iorfino et al., 2017). Given its ease of administration, low cost and brevity it provides a means to measure PD in a standardised way over time and allows for comparability across studies. Recent research of Australian adults (over 25 years) found that those with ‘low’ PD at their initial survey were likely to remain low, providing a stable ‘control’ group (Welsh et al., 2020). Comparatively, those who had ‘high’ distress experienced average changes in K10 scores of 4.7 points over an eight year period (measured every 2 years) (Welsh et al., 2020). This suggests there is utility in conducting multiple assessments over time, to gain an accurate representation of an individual’s mental health (Welsh et al., 2020). This may be particularly useful in adolescence, which is characterised by substantial changes in mental health.

A study of adolescents and young adults found that hippocampal grey matter development may be moderately-highly heritable, with evidence of region-specific environmental influences (Rentería et al., 2014). This provides support for investigating the relationship with mental health to uncover novel targets for intervention, particularly early in development. Recent research has highlighted the benefits of looking at neurobiological markers with PD, at short intervals, in young people. Broadhouse et al. (2019) found that grey matter volume (GMV) in the left hippocampus subregion CA1 was negatively correlated with PD in *N* = 32 12-year-olds. These data were part of the Longitudinal Adolescent Brain Study (LABS), and four-month follow-up analysis of a sub-sample (*n* = 24) showed a significant association between change in PD and changes in left CA1 GMV, whereby reduced K10 scores correlated with increased left CA1 GMV (Broadhouse et al., 2019). Jamieson et al. (2022) examined *N* = 63 LABS 12-year-olds and found sex differences in hippocampal GMV, K10, and sleep quality, with poor sleep quality predicting PD in females only. Further recent research on the ‘first hundred brains cohort’ of LABS participants also found differences in subcortical volumes between sexes and that females experienced greater psychological distress, however, there were no associations found between these measures, by sex (Levenstein et al., 2023). These findings highlight the complex relationships between hippocampus GMV and PD in early adolescence, indicating that additional longitudinal research is needed. Further, it has been recommended that neuroimaging research should consider differences in trajectories of cortical and subcortical grey matter development (between individuals and groups), ideally within narrow age ranges (rather than averages across long intervals), and ensure groups are sex-matched (Barch et al., 2021; Giedd et al., 1999; Gogtay et al., 2006; Herle et al., 2020; Lenroot et al., 2007; Luby et al., 2016; Schmaal et al., 2017b). Therefore, PD scales may be utilised in combination with brain imaging measures to better understand the links between brain changes and likelihood of mental illness in youth.

Thus, this short-interval longitudinal study examined data collected at four timepoints, over a 12-month period, for early adolescents aged 12–13 years from LABS. Data included GMVs for the whole hippocampus bilaterally, as well as scores on a self-reported PD scale (K10) (Kessler et al., 2003). It was hypothesised that: (1) young people with moderate-to-high levels of PD at any point in the year, would have more variability in K10 scores, compared with those who only experienced low PD; (2) changes in PD would be associated with changes in hippocampal GMV; and (3) young people who experienced moderate-to-high levels of PD would exhibit different associations with hippocampal GMV over time, compared with those who only experienced low PD over the year.

## Method

### Study design and participants

Ethics approval was granted by the UniSC Human Research Ethics Committee (Approval A181064). This study utilised self-report and neuroimaging (MRI) data collected over the first year (four timepoints) of LABS (design and recruitment are described elsewhere; (Beaudequin et al., 2020; Boyes et al., 2022)). Written consent was obtained from all parents/caregivers and participants. Assessments were completed with trained researchers at the Thompson Institute (TI), UniSC. The current sample included *N* = 88 participants. Participants (40 female; 7 left-handed) completed a minimum of two timepoints, including both K10 and MRI data over the year, resulting in a total of 275 timepoints of data (an average of 3.14 timepoints per participant), at 3 July 2023; see Table 1 for demographics for all timepoints. These datasets were confirmed to comprise valid scores, within appropriate QC ranges, and excluded any MRI scans impacted by artefact (such as braces or excessive motion).

**Table 1:** Key demographics by timepoint (*N* = 88)

Timepoint	Mean age in years (SD)	Grade 7, *n*	Grade 8, *n*	Grade 9, *n*	Male, *n*	Female, *n*	*N*
1	12.60 (0.31)	77	0	0	44	33	77
2	12.92 (0.32)	48	29	0	43	34	77
3	13.23 (0.32)	11	52	0	33	30	63
4	13.63 (0.32)	1	56	1	32	26	58

#### Inclusion and exclusion criteria

Selection criteria included participants aged 12 years, in their first year of secondary school (Grade 7) and proficient in spoken and written English. Young people were excluded if they: suffered from a major neurological disorder, intellectual disability, or major medical illness; had sustained a head injury (with loss of consciousness more than 30 min); or if they were unable to complete the MRI.

### Measures

#### Self-reported psychological distress (K10)

**Fig. 1:**
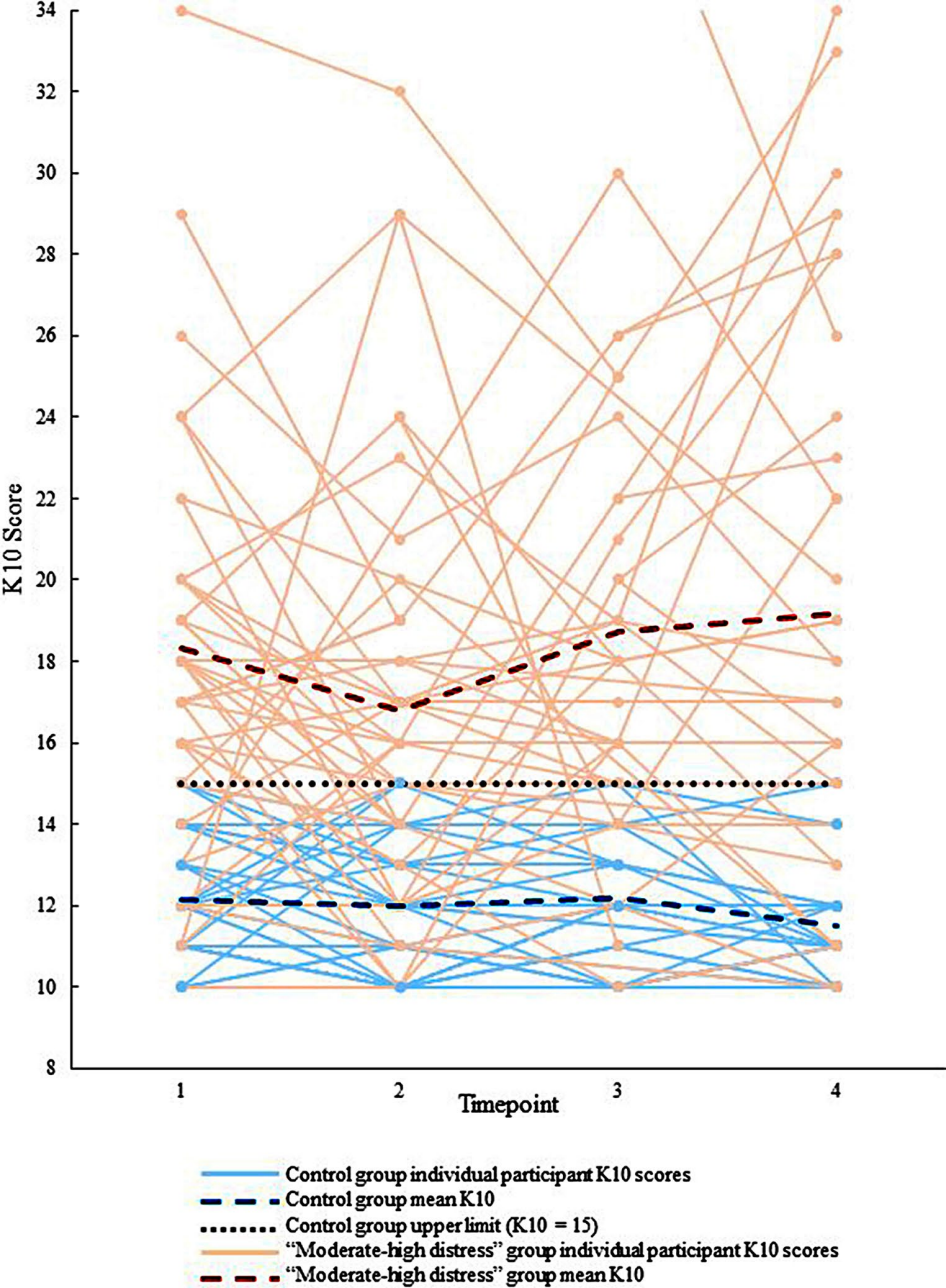
Individual K10 scores TP1-TP4 (*N* = 88) coded by “moderate-high distress” (*n* = 50) and control (*n* = 38) groups

Participants completed the K10 scale, with 10 items answered on a 5-point scale (1 = *none of the time* to 5 = *all of the time*), and an overall score of 10–50. The K10 is a valid measure of PD in adolescents over the previous 30 days, which uses the participant’s total score to identify their level of non-specific PD and likelihood of psychological disorders, particularly depression and anxiety (Andrews & Slade, 2001; Chan & Fung, 2014; Kessler et al., 2003; Lawrence et al., 2015; Sunderland et al., 2011). K10 scores were utilised to group participants for the purpose of the analyses (see Fig. 1; Table 2 and Supplemental Material).

#### Magnetic resonance imaging

Participant MRI brain scans were acquired using a 3-Tesla Siemens Skyra scanner (Erlangen, Germany) with a 64-channel head and neck coil, performed at the Nola Thompson Centre for Advanced Imaging (TI, UniSC). As part of the MRI protocol, 3D whole brain structural imaging was acquired using a T1-weighted magnetization prepared rapid acquisition gradient echo sequence (MP-RAGE: TR = 2200 ms, TE = 1.71 ms, TI = 850 ms, flip angle = 7°, spatial resolution = 0.9 × 0.89 × 0.89 mm, FOV = 208 × 256 × 256, TA = 3:57). T1-weighted images were processed using FreeSurfer’s (V7.2) recon-all pipeline (Dale et al., 1999; Fischl et al., 2002), and normalised using the proportional approach to create adjusted GMVs prior to statistical analysis (O’Brien et al., 2011) (see Supplemental Material for additional information regarding MRI structural analysis). Analyses included the whole hippocampus, for each timepoint, bilaterally.

### Statistical analyses

#### Data screening

Following normalisation of GMV, data were checked for validity and outliers. All primary outcome variables were confirmed to comprise valid scores there were no artefactual outliers. Statistical analyses were carried out using SPSS Statistics for Windows, Version 28 (IBM Corp., Armonk, NY).

#### Generalised estimating equations analyses (GEE)

The mean and 95% confidence interval (CI) of the bilateral whole hippocampal GMV, sex differences, and longitudinal changes (associations with age) were estimated using Generalised Estimating Equations (GEE). We also tested biological sex difference due to previously identified differences in hippocampal grey matter volumes in female and male adolescents in this cohort (Jamieson et al., 2022; Levenstein et al., 2023). In line with previous analyses examining functional brain fingerprinting and PD (Shan et al., 2022a, b) a full GEE model was conducted with the following settings: the participant ID as the subject variable; unstructured working correlation matrix; a linear link function; K10 as the dependent variable; sex as the factor; age at K10 timepoint, whole left hippocampal GMV, whole right hippocampal GMV and euler number as covariates; and chi-square statistics for model effect testing. Euler numbers were used as a covariate to confirm whether MRI data quality was associated with any significant associations with GMV (see Supplemental Material). Output was split by group.

## Results

**Fig. 2:**
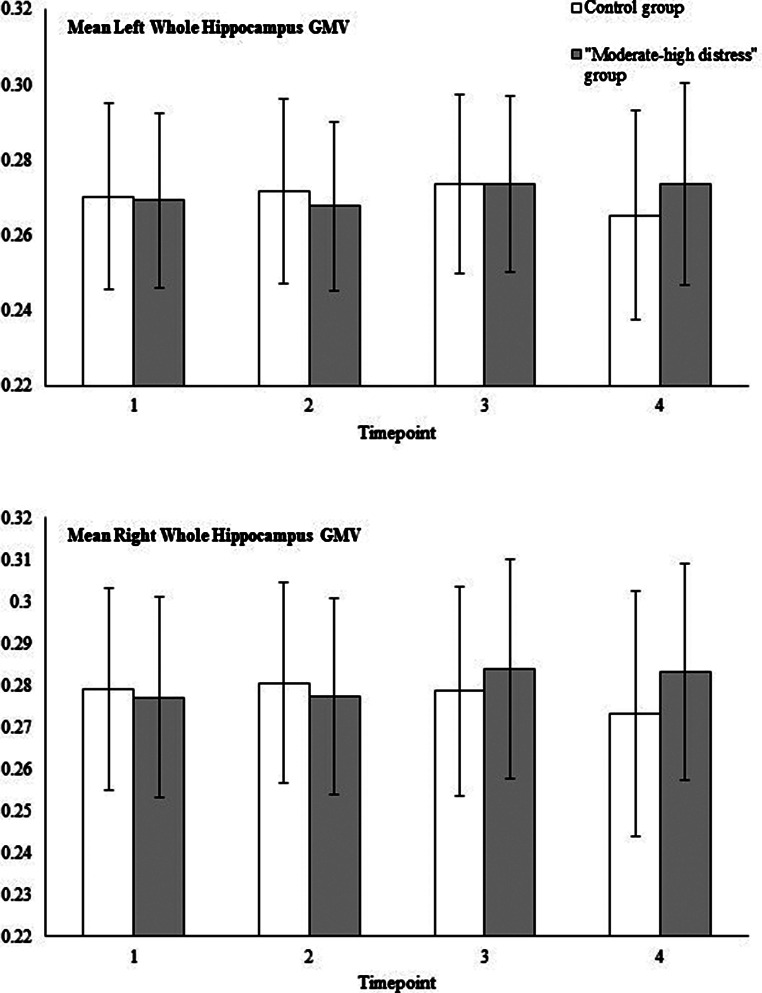
“Moderate-high distress” (*n* = 50) and control (*n* = 38) group means and standard deviations hippocampus GMV (proportionally normalised)

Tables 1 and 2, and Fig. 2 summarise key self-report and imaging variables over time, by group.

**Table 2:** Means and standard deviations for K10 scores and MRI data, by group (*N* = 88)

Measure	“Moderate-high distress” Group (*n* = 50, 26 female, 43 right-handed, 2 ambidextrous)				Control Group (*n* = 38, 14 female, 36 right-handed)			
	T1 M (SD)	T2 M (SD)	T3 M (SD)	T4 M (SD)	T1 M (SD)	T2 M (SD)	T3 M (SD)	T4 M (SD)
K10 score	18.31 (5.29)	16.79 (5.06)	18.71 (5.87)	19.17 (6.84)	12.16 (1.61)	11.97 (1.66)	12.18 (1.52)	11.48 (1.34)
Left whole hippocampus	.269 (0.023)	0.268 (0.023)	0.274 (0.023)	0.274 (0.027)	0.270 (0.025)	0.272 (0.024)	0.274 (0.024)	0.265 (0.028)
Right whole hippocampus	0.277 (0.024)	0.278 (0.023)	0.284 (0.026)	0.283 (0.026)	0.279 (0.024)	0.281 (0.024)	0.279 (0.025)	0.273 (0.029)
eTIV	1456590.53 (140660.82)	1466917.86 (148851.13)	1452031.45 (130495.42)	1460284.30 (154800.41)	1468625.65 (135253.16)	1472171.25 (133062.25)	1472666.69 (124249.94)	1472454.52 (126707.70)
Euler number	-84.93 (32.95)	-81.40 (47.45)	-69.03 (23.35)	-68.97 (29.92)	-84.19 (33.02)	-83.88 (36.34)	-71 (37.24)	-83.57 (36.03)

*Note*: GMV was normalised using the proportional method (ROI/eTIV*100). eTIV = Estimated Total Intracranial Volume (mm^3^). “Moderate-high distress” group K10 scores were > 16 at least once over the course of the year; control group K10 scores were between 10–15 for all four timepoints

### GEE

Associations tested by GEE fitting of GMV and K10 measures at the same timepoint, and in the preceding and subsequent timepoints are summarised in Table 3. Smaller preceding left GMV and larger preceding right GMV were associated with higher subsequent K10 scores in the “moderate-high distress” group, four months later. The control group showed significant co-occurring associations (i.e., at the same timepoint) between GMV and K10 scores. There were no significant associations with age or sex in any of the models. Euler number was also non-significant in both analyses with significant associations between K10 and GMV.

**Table 3:** GEE Wald chi-square associations between K10 scores and hippocampal GMV

GMV	K10 score at preceding timepoint	K10 score at co-occurring timepoint	K10 score at subsequent timepoint
*Control group*			
Whole left hippocampus	3.46	4.43*	1.98
Whole right hippocampus	3.17	5.32*	1.87
Age (in weeks) at K10 timepoint	0.03	0.56	0.46
Sex	0.04	0.80	0.26
*“Moderate-high distress” group*			
Whole left hippocampus	0.39	0.30	10.38**
Whole right hippocampus	0.26	1.17	10.84**
Age (in weeks) at K10 timepoint	3.22	0.02	0.17
Sex	1.49	0.50	0.62

*Note*: Associations between K10 and GMV were estimated using GEE modelling accounting for repeated measures with covariates of sex, age at K10 timepoint, and euler number (i.e. MRI data quality)

*p < .05; **p ≤ .001

## Discussion

This study investigated associations between hippocampal GMV and PD longitudinally, in early adolescence. The dataset was temporally rich and allowed for a novel approach. Our first hypothesis, that young people with “moderate-to-high” levels of PD at any point in the year, would have more variability in K10 scores, compared with those who only experienced low PD, was confirmed. Table 2 shows that in addition to having the expected higher mean K10 scores, the “moderate-high distress” group also had a larger standard deviation when compared to the control group, exhibiting similar patterns to those observed in adults. Recent research in adults found those with ‘low’ PD at their initial survey were likely to remain low, providing a stable ‘control’ group, while those who had ‘high’ distress experienced greater changes in K10 over time (Welsh et al., 2020). Our study has expanded on these results by revealing that in early adolescents, K10 scores at initial visit do not necessarily remain stable over 12 months (see Fig. 1). Furthermore, *n* = 30 out of the *n* = 50 young people in the “moderate-high distress” group had K10 scores ≤ 15 at least once over the year, highlighting the potential for missed intervention opportunities, and utility of multiple measurements of PD, even at short intervals.

Our second and third hypotheses, that: changes in PD would be associated with changes in hippocampal GMV; and that young people who experienced “moderate-to-high” levels of PD would exhibit different associations with hippocampal GMV over time, compared with those who only experienced low PD, were confirmed. Previous LABS research reported that longitudinal GMV increases in the left CA1, were associated with reduced PD (Broadhouse et al., 2019). The current study found different associations between bilateral whole hippocampal GMV and K10 scores over time, by PD group. Smaller preceding GMV and larger preceding right GMV were associated with higher subsequent K10 scores in the “moderate-high distress” group. This association was not found in the control group. In contrast, the control group showed significant co-occurring associations (i.e., at the same timepoint) between GMV and PD. However, this finding may be misleading, as the control group had very small changes in K10 scores (i.e., remained ‘low’) over the year. Thus, the shift in PD for the control group doesn’t have the clinical utility it does in the “moderate-high distress” group. Rather, the observed volume changes probably reflect typical neurobiological development, or may indicate an interaction with different environmental supports or positive experiences during times of distress not present in the “moderate-high distress” group (Miguel et al., 2019).

This study highlights the utility of examining individual trajectories in early adolescence, rather than averages, to provide more fine-grained health information (Herle et al., 2020). Broad group-level averages across time do not necessarily reflect individual trajectories in GMV (Lenroot et al., 2007), and this study found different, subtle trajectories within a narrow age range. Further, hippocampal GMV may fluctuate more than previously observed and may be more state-based, particularly in early adolescence (Schriber et al., 2017). Smaller sample sizes have implications for generalisability, however, useful results can still be garnered by capturing multiple data points of a small sample (such as the approach in this study) (Klapwijk et al., 2021), or employ Bayesian statistical analyses to estimate the likelihood of both a null and alternative hypothesis (Szucs & Ioannidis, 2017). Thus, while the current sample was further reduced by creating sub-groups, the larger volume of data was a strength of this study. Further, there is emerging counter-argument that “bigger” is not necessarily “better”, as large datasets may increase bias, and researchers should aim for a “cost-effective” sample, based on calculations involving effect size, p value, statistical test, power as well as considerations about participants and data type (Kaplan et al., 2014; Serdar et al., 2021).

It is hoped that the approach undertaken here can be replicated in future studies, as it is important to analyse differences between individuals who experience fluctuations into increased PD (compared with those who don’t). Most previous neuroscience research investigating the links between neurobiology and mental health have looked at group-level differences between those with/without a certain diagnosis or prior experience (Schmaal et al., 2017a; Whittle et al., 2013); changes over longer periods of time (Papmeyer et al., 2016; Whittle et al., 2014); or changes over the course of an intervention (Zhang et al., 2018). The current study looked at changes at intervals of four months, in early adolescence and without any specific interventions (beyond what the participants were doing in their normal, everyday life). This adds a unique perspective of patterns of factors associated at the ‘micro-level’ experience of an individual (Wichers, 2014).

People who experience poor mental health have larger fluctuations in subclinical symptoms of mental ill-health and wellbeing. This is supported by others who have noted that depression is a dynamic experience for individuals, marked by momentary states, and there are benefits to incorporating both ‘micro’ and ‘macro’ level information in order to understand disorders of mental health (Wichers, 2014). Thus, future research could combine longitudinal modelling of larger datasets (Guo et al., 2013) with consideration of discreet timepoints to improve our understanding of co-occurring micro-scale, individual-level changes, while also understanding broader group-level trends and differences. There are challenges with grouping individuals based on single timepoints, thus, grouping young people based on whether they passed a threshold of poor mental health at any time during the year provides a novel and useful alternative approach. The inclusion of 275 datasets in analyses goes some way to address concerns of sample size, accuracy of behavioural measurements, and individualisation of findings that can be used to understand broader group-level differences, while also bridging the gap to translation of research to moment-to-moment individual experiences (Wichers, 2014). By considering trajectories and fine-grain longitudinal data, classifications and experiential information is more able to be identified and utilised in developing an understanding of potential biological, psychological and environmental needs and differences.

As there are no comparable studies examining the same adolescents every four months, future research in this sample may uncover further information about GMV changes in this cohort. While sex did not impact results, there may be other co-occurring factors that could interact with brain structure, such as wellbeing or social environment (Schriber et al., 2017), puberty (Lenroot et al., 2007), COVID-19 infection (Douaud et al., 2022) or COVID-19 worry (Jamieson et al., 2021). Results should be replicated in other early adolescent samples, using comparable analysis techniques to see whether results are upheld (Pruessner et al., 2000). Recent research has found links between brain function in the posterior and anterior hippocampus in adults, with symptoms of anxiety and PTSD (Abdallah et al., 2017; Chaposhloo et al., 2023; Satpute et al., 2012). Thus, future research could examine whether there are similar associations between hippocampus sub-structures and sub-clinical anxiety and depressive symptoms. Future research from LABS should also determine whether the patterns observed in this first year of the study are upheld over time.

## Conclusion

Our study found that young people aged 12–13 years had different experiences of PD, and different associations between hippocampal GMV and PD, depending on whether they experienced consistently “low” or “moderate-to-high” PD at any point during a year. To our knowledge, this is the first time this has been observed in a study investigating subclinical measures of mental health and GMV in a community cohort of adolescents.

### Electronic supplementary material

Below is the link to the electronic supplementary material.

Supplementary Material 1

## Data Availability

The datasets generated and analysed during the current study are not publicly available due the fact that they constitute an excerpt of research in progress but are available from the corresponding author on reasonable request.

## Code availability

Structural analyses were conducted using FreeSurfer’s V7.4.0 recon-all pipeline.
