# Supplementary material for: A short-interval longitudinal study of associations between psychological distress and hippocampal grey matter in early adolescence
Source: Brain Imaging Behav. 2024 Jan 13;18(3):519–28. doi: 10.1007/s11682-023-00847-6 (PMC11222233; doi:10.1007/s11682-023-00847-6)
Supplement: Supplementary file 1 — Supplementary Material 1 [file 11682_2023_847_MOESM1_ESM.docx]

**Supplemental Material**

**Measures**

#### *Magnetic Resonance Imaging (MRI)*

**Structural Analysis**. T1-weighted images were processed using FreeSurfer’s (V7.4.0) recon-all pipeline, which parcellates the brain into cortical and subcortical regions by reconstructing the cortex and conducting volumetric segmentation (Dale et al., 1999; Fischl et al., 2002). FreeSurfer has been utilised in longitudinal and cross-sectional studies of adolescent brains and uses reliable and valid surface-based modelling to semi-automatically parcellate the brain and calculate GMV for specific regions (Fischl, 2012; Fischl et al., 2002). For the current study, the longitudinal recon-all pipeline was implemented to create CROSS (i.e., cross-sectional), BASE (i.e., within-subject), and LONG (i.e., longitudinal) analyses for each participant (Reuter et al., 2012). Processing was run using Freeesurfer 7.4.0 that had been packaged in singularityCE (version 3.8.0). The processing was completed on the UniSC HPC system using PBS (version 20.0.0) running Ubuntu 20.04 connected to a CEPH cluster (version 15.2.13). Whole hippocampal GMV and estimated total intracranial volumes (eTIV) were extracted from the longitudinal subcortical segmentation (‘aseg’) (Fischl et al., 2002). Data was normalised using the proportional approach to create adjusted GMVs prior to statistical analysis (O'Brien et al., 2011).

Following extraction of MRI data, Euler numbers were calculated for all 275 MRI scans using the below formula:

***Euler number = 2 – 2 x total FreeSurfer CROSS brain surface hole number***

Euler numbers provide an indication of cortical surface topology and have been used in neurodevelopment studies as a measure of image quality (Dale et al., 1999; Rosen et al., 2018). A perfect surface with no holes would have a Euler number of two, however, in adolescent imaging studies, it has been previously reported that values can be as low as -380 and may mediate apparent relationships between grey matter and age (Rosen et al., 2018). These numbers have been related to manual ratings, with potential additional benefits as they provide a quantitative metric that does not introduce potential error as a result of differences in researcher manual edits (Monereo-Sánchez et al., 2021; Rosen et al., 2018). Raw scans and FreeSurfer segmentations were checked for participants with the most extreme Euler numbers and confirmed to be valid and accurate datasets.

#### *Self-reported Psychological Distress (K10)*

**Group classification.** K10 scores across TP1-TP4 were used to classify participants (see Figure 1). The “low distress” group (*n*=38; 14 female; 36 right-handed) had K10 scores between 10-15 for all four timepoints (that is, they did **not** have *moderate psychological distress or higher* at **any** point during that year). This group is referred to as the ‘control’ group in the remainder of this paper. The “moderate-high distress” group (*n*=50; 26 female, 43 right-handed, 2 ambidextrous) had K10 scores that were 16 or higher at least once over the course of the year. Cut-offs for groups were based on the Australian Bureau of Statistics Information Paper which categorises K10 scores of 10-15 as ‘low’, 16-21 ‘moderate’, 22-29 ‘high’ and 30-50 ‘very high’(Andrews & Slade, 2001; Australian Bureau of Statistics, 2012). There were no significant differences between groups on sex or handedness (Chi-square Fisher’s Exact Test *p* > .05).

References

Andrews, G., & Slade, T. (2001). Interpreting scores on the Kessler Psychological Distress Scale (K10). *Australian and New Zealand Journal of Public Health*, *25*(6), 494-497. <https://doi.org/10.1111/j.1467-842X.2001.tb00310.x>

Australian Bureau of Statistics. (2012). 4817.0.55.001 - Information Paper: Use of the Kessler Psychological Distress Scale in ABS Health Surveys, Australia, 2007-08. <https://www.abs.gov.au/ausstats/abs@.nsf/lookup/4817.0.55.001chapter92007-08>

Dale, A. M., Fischl, B., & Sereno, M. I. (1999). Cortical surface-based analysis. I. Segmentation and surface reconstruction. *Neuroimage*, *9*(2), 179-194. <https://doi.org/10.1006/nimg.1998.0395>

Fischl, B. (2012). FreeSurfer. *Neuroimage*, *62*(2), 774-781. <https://doi.org/10.1016/j.neuroimage.2012.01.021>

Fischl, B., Salat, D. H., Busa, E., Albert, M., Dieterich, M., Haselgrove, C., van der Kouwe, A., Killiany, R., Kennedy, D., Klaveness, S., Montillo, A., Makris, N., Rosen, B., & Dale, A. M. (2002). Whole brain segmentation: automated labeling of neuroanatomical structures in the human brain. *Neuron*, *33*(3), 341-355. <https://doi.org/10.1016/s0896-6273(02)00569-x>

Monereo-Sánchez, J., de Jong, J. J. A., Drenthen, G. S., Beran, M., Backes, W. H., Stehouwer, C. D. A., Schram, M. T., Linden, D. E. J., & Jansen, J. F. A. (2021). Quality control strategies for brain MRI segmentation and parcellation: Practical approaches and recommendations - insights from the Maastricht study. *Neuroimage*, *237*, 118174. <https://doi.org/https://doi.org/10.1016/j.neuroimage.2021.118174>

O'Brien, L. M., Ziegler, D. A., Deutsch, C. K., Frazier, J. A., Herbert, M. R., & Locascio, J. J. (2011). Statistical adjustments for brain size in volumetric neuroimaging studies: Some practical implications in methods. *Psychiatry Research*, *193*(2), 113-122. <https://doi.org/10.1016/j.pscychresns.2011.01.007>

Reuter, M., Schmansky, N. J., Rosas, H. D., & Fischl, B. (2012). Within-subject template estimation for unbiased longitudinal image analysis. *Neuroimage*, *61*(4), 1402-1418. <https://doi.org/https://doi.org/10.1016/j.neuroimage.2012.02.084>

Rosen, A. F. G., Roalf, D. R., Ruparel, K., Blake, J., Seelaus, K., Villa, L. P., Ciric, R., Cook, P. A., Davatzikos, C., Elliott, M. A., Garcia de La Garza, A., Gennatas, E. D., Quarmley, M., Schmitt, J. E., Shinohara, R. T., Tisdall, M. D., Craddock, R. C., Gur, R. E., Gur, R. C., & Satterthwaite, T. D. (2018). Quantitative assessment of structural image quality. *Neuroimage*, *169*, 407-418. <https://doi.org/https://doi.org/10.1016/j.neuroimage.2017.12.059>
